# Computer Navigation vs. Conventional Overlay Methods in Direct Anterior Total Hip Arthroplasty: A Single Surgeon Experience

**DOI:** 10.7759/cureus.29907

**Published:** 2022-10-04

**Authors:** Parker Goodell, Sean Ellis, Brent Kokobun, Holly Wilson, Robert C Kollmorgen

**Affiliations:** 1 Orthopedic Surgery, University of California San Francisco, Fresno Medical Education Center, Fresno, USA; 2 Orthopedics, Orthopedic One, Columbus, USA; 3 Orthopedics, University of California San Francisco, Fresno Medical Education Center, Fresno, USA; 4 Orthopedics, University of California San Francisco, Fresno, USA; 5 Hip Preservation and Sports Medicine, University of California San Francisco, Fresno, USA

**Keywords:** radiation, efficiency, total hip arthroplasty, direct anterior, technology, navigation, computer navigation

## Abstract

Background: The use of computer navigation (CN) is expanding in direct anterior (DA) total hip arthroplasty (THA). In this study, we investigated the use of a noninvasive, fluoroscopic-based, CN technology suite on operative outcomes in a single surgeon DA THA practice.

Hypothesis: Computer-navigated DA THA decreases leg length discrepancy (LLD) variation and fluoroscopic radiation dose without adding operative time compared to the traditional overlay (OL) technique.

Methods: A retrospective review was performed on a total of 109 primary DA THA patients, with 58 in the CN and 51 in the OL group. Outcome metrics were postoperative LLD, radiation dose per case, and operative time. Statistical analysis was completed with Mann-Whitney U tests for differences between the means for LLD, radiation dose, and operative time.

Results: No difference was observed in postoperative LLD between the CN (average: 1.8 mm) and OL (average: 1.9 mm) groups (p = 0.458). A significant reduction in average radiation dose (mGy) per case within the CN group (8.17 ± 6.09 mGy) compared to the OL group (13.17 ± 7.75 mGy) (p < 0.02) was observed. The average operative time in the CN group was 80 ± 18 minutes compared to 120 ± 32 minutes in the OL group (p < 0.01).

Conclusion: There was no difference in LLD between the two groups. The addition of CN into a DA THA practice decreased both average radiation dose and operative time when compared to the standard OL technique.

## Introduction

The direct anterior (DA) approach has become an increasingly popular approach to total hip arthroplasty (THA), with the recent American Association of Hip and Knee Surgeons (AAHKS) annual meeting survey showing use in 45% of all THA, increased from 40% in 2018 [1]. With this shift in technique, a large focus has been placed on improving outcomes, including arthritic pain relief and leg length discrepancy (LLD). In particular, LLD after THA has been associated with poor patient-reported outcomes, gait abnormalities, aseptic loosening, new onset back pain, nerve palsy, and a common source of litigation [2-7].

Traditionally, the conventional overlay (OL) technique employs printed intraoperative radiographs and OL templates to determine appropriate leg length and offset [8]. More recently, computer navigation (CN) and robotics have been developed as adjuncts to maximize native hip mechanics to improve surgical outcomes, reduce dislocation rates, and maximize patient satisfaction [9-12]. These technologies include a variety of intraoperative CN systems that have been used to provide real-time, data-driven feedback on prosthetic placement in the operating room [9,10,13,14].

The use of CN in THA, however, has demonstrated mixed results in providing clear, consistent benefits. A meta-analysis by Xu et al. (2014) found decreased LLD and improved acetabular cup placement in 1071 hips with CN [14]. However, a large Medicare database study by Montgomery et al. (2019) comparing over 60,000 THA patients did not show a significant reduction in postoperative dislocations or revision surgery between navigated and conventional THA [10]. A review of the literature by Rajpaul et al. (2018) showed an overall improvement in restoring leg lengths compared to conventional techniques, but without strong functional benefits in the long term [15]. Despite these differences, the use of CN in THA has overall demonstrated the potential to streamline the procedure via reducing trialing, interruptions for repeat fluoroscopy, and decreased operative times compared to conventional techniques [13,16].

This study proposes to evaluate the effect of the addition of CN to a DA practice. We hypothesize that computer-navigated DA will decrease LLD, operative times, and fluoroscopic radiation dosing compared to the traditional OL technique.

## Materials and methods

A retrospective review was performed on a consecutive series of patients who had undergone primary DA THA (Current Procedural Terminology (CPT) code 27130) by the senior author between July 2017 and October 2019. The senior author (RCK) is a fellowship-trained, orthopedic surgeon with >1000 DA THA experience prior to this cohort. All surgeries took place in the presence of orthopedic surgery residents. This study received institutional review board (IRB) approval from the University of California San Francisco (UCSF-IRB 18-26040). Inclusion criteria included patients undergoing a primary DA THA performed by the senior author. Exclusion criteria included revision THA, an approach other than DA, conversion arthroplasty, and missing or inadequate radiographs. No cementation was used during implantation. A total of 109 primary DA THAs were included in this review, with 58 hips in the CN group and 51 in the traditional OL group. This is a consecutive series of patients before and after the implementation of CN, there was no return to the OL technique nor was there any patient selection or randomization into OL or CN. Due to the consecutive nature of the study, the patients were not matched into either group. Outcome metrics of the study were postoperative LLD, operative time, and radiation dose (mGy) per case.

**Figure 1:**
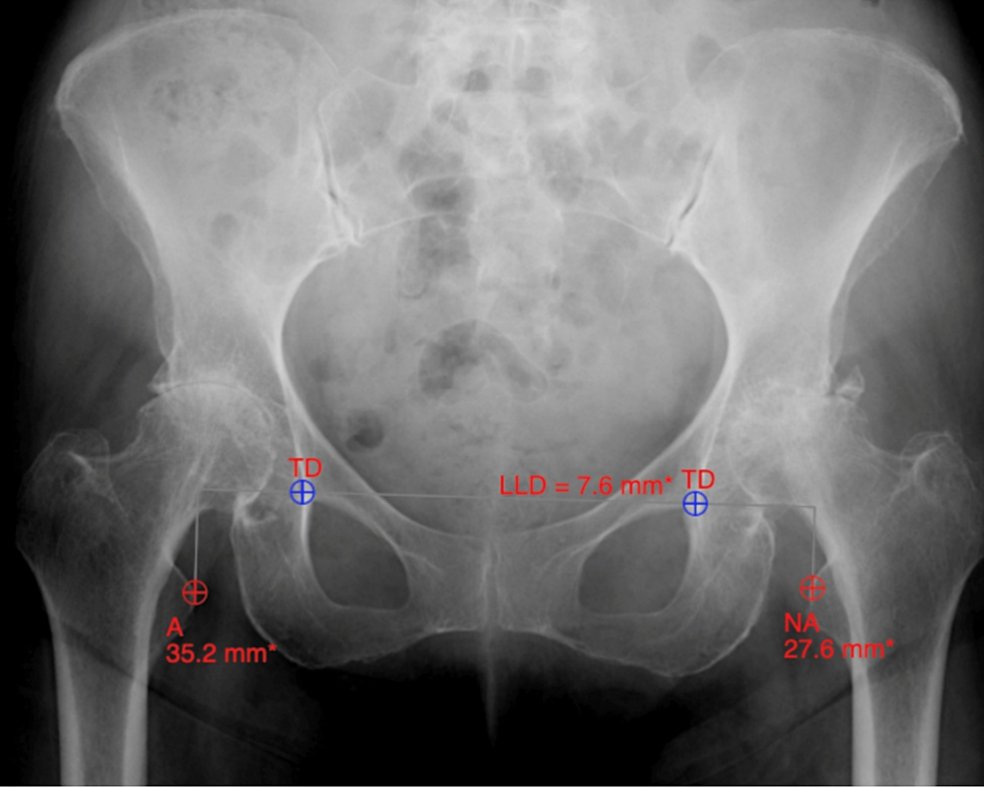
Determining leg length discrepancy preoperatively using the trochanteric technique. TD: teardrop; LLD: leg length discrepancy.

LLD was assessed by two independent reviewers (two senior orthopedic surgery residents) via the trochanteric technique. Briefly, the teardrops were marked bilaterally on each anteroposterior pelvis film and a horizontal line was drawn connecting the two points [17]. Next, the distance from the most prominent portion of the lesser trochanter to the horizontal teardrop line was measured to approximate each leg length and calculate the difference for each film (Figure 1). This measurement was performed twice by each observer in random order and two weeks apart to limit recall bias. Measurements were taken from radiographs at the most recent preoperative visit and from the first postoperative clinic appointment. Abduction angles were calculated by CN software intraoperatively for computer-navigated hips and were placed between 40° and 45° using OL transparency angles. Tönnis grade (osteoarthritis) and Crowe classification (dysplasia) were determined for each patient based on preoperative radiographs by the senior author. Outcomes were measured as intraoperative or postoperative complications within 90 days requiring a reoperation.

Operative time was determined using the OR log in the hospital electronic medical record (EMR) and calculating minutes between procedure start time and end time. This was documented by hospital protocol by the circulating OR nurse. Radiation dosage was collected from the fluoroscopic report in the EMR and reported in mGy.

An a priori power analysis was performed and determined that 31 patients per group were needed to obtain p < 0.05 with 1-b = 0.8. Statistical analysis was performed using JASP (version 0.14.1; University of Amsterdam, Amsterdam, The Netherlands) and Microsoft Excel (Microsoft Corporation, Redmond, WA) with the Mann-Whitney U test for differences between the mean for the cohort’s LLD, OR time, and radiation dose. Categorical variables were analyzed utilizing Pearson’s chi-squared test. Inter- and intraobserver coefficients were calculated for LLD measurements performed by the senior residents. Intraclass correlation coefficients (ICC) with a 95% CI were calculated for intra- and interobserver reliability and interpreted as minimal (<0.2), poor (0.2 to <0.4), moderate (0.4 to <0.6), strong (0.6 to ≤0.8), and excellent (>0.8). Bland-Altman plots were utilized to analyze the agreement between the measurements for all observations.

**Figure 2:**
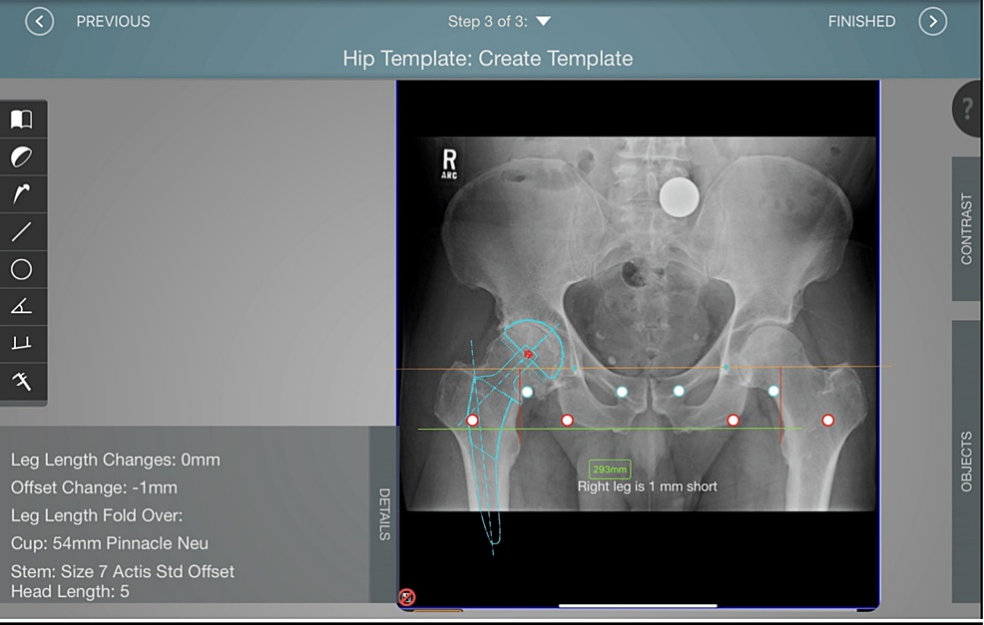
Screenshot of Velys™ preoperative templating with estimated implant size, offset, and leg length changes.

**Figure 3:**
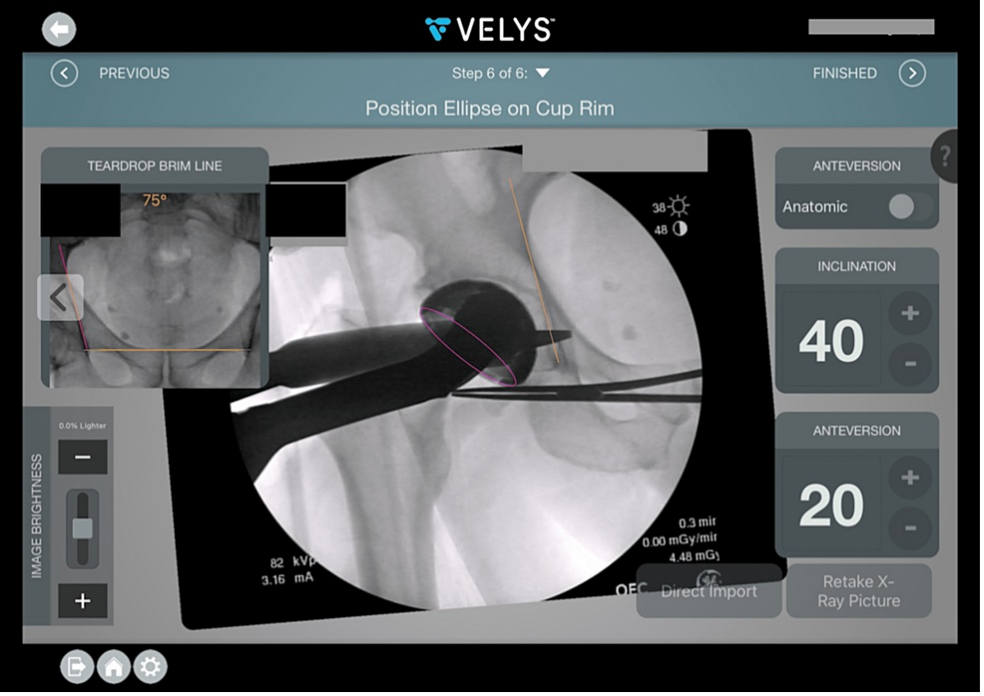
Screenshot of Velys™ intraoperative with acetabular cup trial based on pelvic landmarks determined from initial pelvis radiograph.

**Figure 4:**
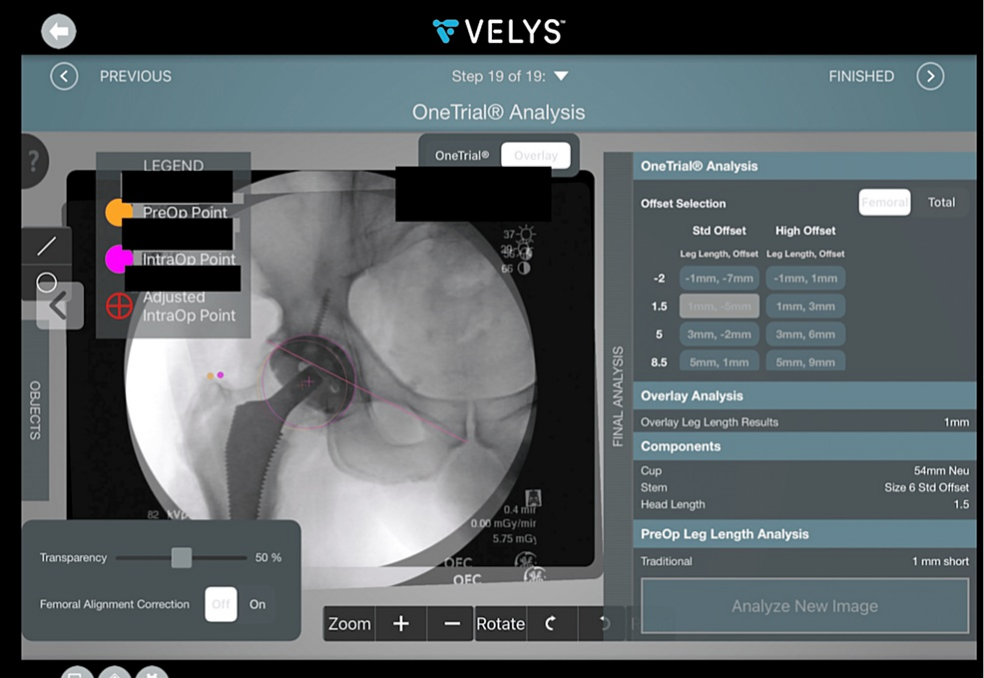
Screenshot of Velys™ intraoperative system with OneTrial analysis and various femoral head sizes and leg length/offset values for each.

The navigation tool utilized was Velys, formerly JointPointTM (Synthes, Palm Beach Gardens, Florida), which is a preoperative and intraoperative navigation system that allows for estimation of offset, LLD, and acetabular cup placement based on intraoperative fluoroscopy. This software was used in preoperative templating, intraoperatively by the lead author as a routine part of the practice and was consecutively utilized for the CN cohort of patients (Figure 2). Additionally, this software utilizes intraoperative fluoroscopic images with user-set reference points to provide an assessment of the LLD, offset, and abduction angle of given hardware (Figures 3, 4) [18].

The OL cohort was performed at the same institution prior to the implementation of computer-navigated software. The OL technique consisted of using printed fluoroscopic radiographs with transparent paper with various implants to assess the offset, LLD, and abduction angle of the acetabular cup [8].

## Results

A total of 109 patients met the inclusion criteria. The CN group consisted of 58 patients and the average age at the time of surgery was 61 ± 1.3 years, while the OL group consisted of 51 patients and the average age was 62 ± 12 years (p = 0.28) (Table 1). The groups were found to be homogeneous in this consecutive cohort as there was no observed difference in preoperative age, sex, BMI, race, or laterality between OL and CN (p > 0.05). There was no difference in preoperative severity of osteoarthritis (Tönnis grade, p = 0.33) or hip dysplasia (Crowe, p = 0.81) (Tables 2, 3).

**Table 1:** Demographic table for navigated and overlay cohorts. Age represented in years.

Navigated (n = 58)				Overlay (n = 51)			
	Mean	SD	Range	Mean	SD	Range	P-value
Age	61	1.37	38-86	62	12.4	34-87	0.28
Sex	36 (62%) female and 22 (38%) male			23 (46%) female and 27 (54%) male			0.09
BMI	29.22	4.8	19.5-44	29.84	4.4	20-40	0.07
Race	51 (88%) Caucasian	7 (12%) Hispanic		32 (64%) Caucasian	11 (22%) Hispanic	7 (14%) other	0.08
Side	25 (43%) left	31 (53%) right	2 (4%) bilateral	19 (38%) left	31 (62%) right		0.48

**Table 2:** Frequency table for Tönnis grade.

Method	Tönnis grade (0-3)					
	0	1	2	3	Total	X^2^ for intergroup difference
Navigated	0	1 (1.7%)	7 (12.1%)	50 (86.2%)	58	p = 0.33
Overlay	0	0	3 (5.9%)	48 (94.1%)	51	

**Table 3:** Frequency table for Crowe classification.

	No dysplasia or Crowe classification (I-IV)						
	Normal	Crowe I	Crowe II	Crowe III	Crowe IV	Total	X^2^ for dysplasia presence
Navigated	49 (84.5%)	7 (12.1%)	1 (1.7%)	1 (1.7%)	0	58	p = 0.81
Overlay	36 (70.6%)	9 (17.6%)	1 (2.0%)	5 (9.8%)	0	51	

The average operative time was 120 ± 32 minutes in the OL cohort and 80 ± 18 minutes in the CN cohort (p < 0.01) (Table 4). EBL and radiation dose were 485 ± 215 mL and 13.17 ± 7.75 mGY in the OL group and 405 ± 172 mL (p = 0.036) and 8.17 ± 6.09 mGY (p < 0.02) in the CN group, respectively. The CN group had an average abduction angle of 40.5 ± 1 degrees and an inclination of 21 ± 2.4 degrees. In the OL cohort, the abduction angle was between 40 and 45 degrees.

**Table 4:** Operative outcome measures EBL: estimated blood loss in milliliters; mGY: milligray; LLD: leg length discrepancy; version represented in degrees. Significant p-values in bold.

	Navigated (n = 58)			Overlay (n = 51)			P-value
	Mean	SD	Range	Mean	SD	Range	
EBL	405	172	150-1000	485	215	100-1100	0.036
Time (minutes)	80	18	59-147	120	32	74-218	<0.01
Radiation dose (mGy)	8.17	6.09	0.33 to 39	13.17	7.75	3.5 to 40	<0.002>
Pre-OP LLD	-2.22	4.8	-13 to 10	-3.6	6.8	-23.9 to 17.4	0.222
Post-OP LLD	1.93	5.9	-6.28 to 26	1.7	7.8	-23 to 21	0.458
Abduction	40.5	1	38-45	40-45	N/A	N/A	
Version	21	2.4	15-25	N/A	N/A	N/A	

**Figure 5:**
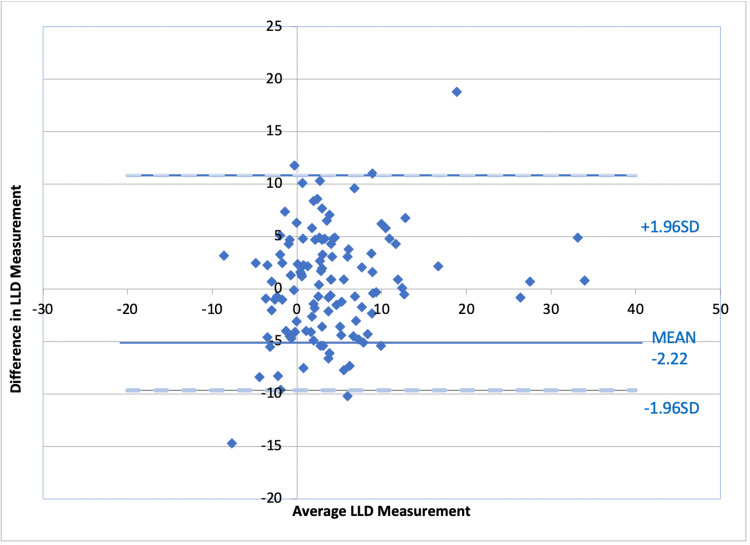
Bland-Altman plot for agreement on leg length discrepancy (LLD) measurements.

Preoperative LLD was -3.6 cm for the OL group and -2.2 cm for the CN group when compared to the contralateral leg with no significant difference (p < 0.220). Postoperative LLD had an average lengthening of 1.9 mm in the CN group and 1.8 mm in the OL group (p = 0.458) (Figure 5). Interobserver reliability for the postoperative LLD radiographic measurements was r = 0.81 (CN) and 0.73 (OL) (Table 5). Intraobserver reliability for postoperative LLD measurement was r > 0.85 for both reviewers in both CN and OL groups (Table 6).

**Table 5:** Interobserver leg length discrepancy r-coefficients CN: computer navigation; OL: overlay technique; LLD: leg length discrepancy. Values are represented as r-values.

	r
Preoperative LLD CN	0.85
Postoperative LLD CN	0.81
Preoperative LLD OL	0.62
Postoperative LLD OL	0.73

**Table 6:** Intraobserver leg length discrepancy r-coefficients CN: computer navigation; OL: overlay technique; LLD: leg length discrepancy. Values are represented as r-values.

	Preoperative LLD CN	Postoperative LLD CN
Resident 1	0.71	0.86
Resident 2	0.86	0.91​
	Preoperative LLD OL	Postoperative LLD OL
Resident 1	0.91	0.9
Resident 2	0.87	0.86​

There were no significant differences in observed complications in intraoperative nor the 90 days following the procedures (Table 7). Intraoperatively, the OL group observed two calcar fractures treated with cerclage wiring and no intraoperative complications in the CN group. In the 90 days following the procedure, one patient in the OL group sustained a femur fracture following a fall on postoperative day 14. Three patients in the OL and four in the CN sustained a suspected infection (p = 0.19). One patient in each group sustained a dislocation event. One patient in the OL group had a frank dislocation at eight weeks post surgery and one in the CN sustained a dislocation from aseptic loosening of the acetabulum at seven weeks post surgery (p = 0.88).

**Table 7:** Complications.

	Overlay	Computer navigated	P-value
Intraoperative			
Calcar fracture	2 (4%)	0	-
90-days postoperative			
Fall with a femur fracture	1 (2%)	0	-
Suspected infection	3 (6%)	4 (6.8%)	0.19
Dislocation	1 (2%)	1 (1.7%)	0.88

## Discussion

Our study shows that, in a consecutive series of patients, there was no significant difference in postoperative LLD compared to the conventional OL technique. We observed a significant reduction in OR time and radiation dose with the addition of CN to an established DA THA practice. While the primary outcome of THA is pain relief, it has become increasingly important to identify technologies that maximize efficiency, improve secondary outcomes, and optimize additional costs. In our practice, the addition of CN proved to achieve these goals.

There are a variety of CN technologies available for supplementation of arthroplasty surgeries. These products include robotic assisting arms using spatial arrays temporarily drilled into the femur and pelvis, preoperative CT mapping, and intraoperative fluoroscopy. We propose that a benefit of the current system utilized by the senior author was its noninvasive nature in employing solely the use of intraoperative fluoroscopy, which avoided significant preoperative radiation from a CT scan or insertion of temporary transosseous pins for spatial tracking. While transosseous pin sites have a very low rate of complication, recently found at 0.16% per pin site, the technique does involve additional incisions and instrumentation for the patient [19].

These technologies have shown varied results with respect to patient outcomes, efficiency, and reduction in complications. A recent large Medicare database study comparing CN THA to conventional THA found no decrease in dislocation rate but did demonstrate an increase in periprosthetic fractures and revision THA at 30 days with computer assistance [10]. A similar larger Medicare database study had conflicting findings with a decrease in dislocation rate and acetabular aseptic loosening with computer guidance [9]. There was no difference in femoral component aseptic loosening or periprosthetic joint infection at any time point. Finally, a National Readmission Database (NRD) found that computer-assisted THA had 12% reduced odds of 90-day complications compared to conventional THA [11]. Our findings are in line with the current literature with no significant difference in observed 90-day complications. These large database studies should be interpreted with caution given their structural vulnerability to unknown confounders and inability to attribute causality.

To our knowledge, this represents the first study showing a reduction in radiation dose using CN compared to conventional THA. McArthur et al. reported the first series of average radiation doses over 56 patients in DA THA using intraoperative fluoroscopy [20]. They found no association with BMI, acetabular screw number, or case length to significantly impact dosage. The fluoroscopic radiation dosage in DA THA, in smaller studies, has not been shown to be of significant exposure to either patient or surgeon [21]. Despite the relatively negligible result of radiation exposure in fluoroscopic-assisted DA THA, any reduction in radiation dosage to a surgeon's cumulative lifetime dosage should be considered a benefit.

Our study found a significant and substantial reduction in operative time with the adoption of CN in a DA THA workflow. While these findings are from a single surgeon's experience, they represent the only significant alteration in an established DA THA practice with gains in efficiency. The literature on CN THA has demonstrated mixed results with respect to the effect on OR times. A larger proportion of studies have found no difference in time or slightly increased time with the implementation of CN [12,22-25]. Our study aligns with a smaller minority of examples in the literature of decreased OR times with CN adoption [13]. Given the wide variety and proprietary modalities of what defines a computer-assisted THA, it is difficult to generalize both outcome and efficiency studies.

Another aspect of consideration is the potential cost savings of decreasing operative time with our use of CN. A recent financial analysis found that the cost of an operating room minute, based on the fiscal year 2014 data in California acute care hospitals, was $36-$37 [26]. While the use of the CN technology does come with an additive cost to the case, $500 at our institution, this may be offset by the overall operative time saved both in terms of minute cost and the potential for a higher volume of cases performed each day and the billable navigation CPT code 0054T. Our study found a decrease on average of 40 minutes per case, representing a savings of nearly $1440-$1460 in operating room time.

Limitations

This study has several limitations. Our cohort represents a single surgeon with an experienced DA THA practice and workflow, which limits the generalizability of our result. However, based on the consecutive nature of the series, we feel that our outcomes are fair representations of improvements in care that temper significant bias, as our data were collected from standard EMR sources, and measurements showed high inter- and intraobserver reliability. Another limitation to this CN system is that it requires a trained individual, such as the sales representative in our series, to operate the computer terminal and select anatomic landmarks on imaging. This study also represents the intraoperative evaluation of the effectiveness of CN implementation with a limited assessment of long-term patient report outcomes. Lastly, we did not assess specific acetabular cup placement comparison between groups nor attempt to validate CN-stated cup abduction and version angles.

## Conclusions

In conclusion, our study demonstrates the effect of adding a CN system to a DA THA workflow. Through a single surgeon retrospective consecutive series, we demonstrated an increase in operative efficiency and a decrease in radiation dosage compared to traditional OL techniques with no significant difference in postoperative LLD. This study suggests that the addition of a fluoroscopy-driven CN system into an established arthroplasty practice has the potential to contribute to a more efficient workflow that may translate into cost savings via reduced operative time and increased case volume.
